# Applying Statistical and Complex Network Methods to Explore the Key Signaling Molecules of Acupuncture Regulating Neuroendocrine-Immune Network

**DOI:** 10.1155/2018/9260630

**Published:** 2018-01-29

**Authors:** Kuo Zhang, Xin-meng Guo, Ya-wen Yan, Yang-yang Liu, Zhi-fang Xu, Xue Zhao, Jiang Wang, Yi Guo, Kai Li, Sha-sha Ding

**Affiliations:** ^1^Research Center of Experimental Acupuncture Science, Tianjin University of Traditional Chinese Medicine, Tianjin, China; ^2^School of Electrical Engineering and Automation, Tianjin University, Tianjin, China; ^3^Acu-Moxibustion and Tuina Department, Tianjin University of Traditional Chinese Medicine, Tianjin, China; ^4^College of Traditional Chinese Medicine, Tianjin University of Traditional Chinese Medicine, Tianjin, China; ^5^Department of Gynaecology, First Teaching Hospital of Tianjin University of Traditional Chinese Medicine, Tianjin, China; ^6^Department of Acupuncture and Moxibustion, Tianjin Nankai Hospital, Tianjin, China

## Abstract

The mechanisms of acupuncture are still unclear. In order to reveal the regulatory effect of manual acupuncture (MA) on the neuroendocrine-immune (NEI) network and identify the key signaling molecules during MA modulating NEI network, we used a rat complete Freund's adjuvant (CFA) model to observe the analgesic and anti-inflammatory effect of MA, and, what is more, we used statistical and complex network methods to analyze the data about the expression of 55 common signaling molecules of NEI network in ST36 (Zusanli) acupoint, and serum and hind foot pad tissue. The results indicate that MA had significant analgesic, anti-inflammatory effects on CFA rats; the key signaling molecules may play a key role during MA regulating NEI network, but further research is needed.

## 1. Introduction

Acupuncture is a physical therapy of preventing or treating diseases by inserting needle into specific acupoints. As a kind of nonspecific physical stimulus, the effects of acupuncture are mediated by the regulatory systems in the body. This determines that the basic way of acupuncture effect is regulating the body's condition, with the characteristics of whole regulation.

Researches have shown that the body's inherent regulatory system is neuroendocrine-immune (NEI) network, including nervous system, endocrine system, and immune system, which is the biological basis to maintain the body's homeostasis. The three systems share common signaling molecules and their affiliated receptors, including some neuropeptides, neurotransmitters, cytokines, and hormones, and their receptors [1]. The cells in each system can secrete these signal molecules and at the same time the cells' surface has the moleculars' receptors. Hence, the common signaling molecules and their receptors constitute the molecular structural foundation of NEI network, being responsible for information communication and transmission between the three systems [1, 2].

Some researches had demonstrated that acupuncture could regulate one system of the NEI network [3–6], in which most focus on the nervous system or immune system, but little studies pay attention to the regulatory effect of acupuncture on the whole NEI network [7]. Therefore, in order to reveal the regulatory effect of acupuncture on NEI network, in this study, we analyzed 55 common signaling molecules of NEI network in serum, supernatants from the ST36 acupoint, and hind foot pad tissue in rats with inflammatory pain after acupuncture treatment and further explore the possible key signaling molecules during acupuncture modulating the NEI network by statistical method. Moreover, in order to characterize the interaction between the common signaling molecules and identify the signaling molecules which play a major role (key signaling molecules) in the NEI network, we applied complex network method for further analysis, which is a powerful tool to solve such network problems [8, 9], and hope to provide ideas and methods for fully revealing the mechanism of acupuncture.

## 2. Materials and Methods

### 2.1. Animal Preparation

The experiments were performed on male Wistar rats (weight: 180 ± 20 g) obtained from the Institute of Hygiene and Environmental Medicine, Academy of Military Medical Sciences, PLA (License number SCXK (army) 2009-003). Rats were housed in animal cages under a 12-h light/dark cycle with food and water available ad libitum for 1 week. All animal procedures in this study were performed according to the International Guide for the Care and Use of Laboratory Animals and were approved by the Animal Ethics Committee of Tianjin University of Traditional Chinese Medicine in China (TCM-LAEC2012010).

### 2.2. Experimental Design

The rats were randomly divided into the following groups: (1) normal saline (NS) group: with normal saline injection, (2) CFA group: with CFA injection, and (3) CFA + manual acupuncture (MA) group: with CFA injection and MA manipulation.

### 2.3. Inflammatory Pain Model

Rats per group were injected with either 0.1 mL CFA (Sigma, USA) or NS in the plantar surface of the right hind paw to induce intraplantar inflammation [10].

### 2.4. Measurement of Thermal Hyperalgesia

Thermal hyperalgesia was assessed by hind paw withdrawal latency (PWL) to a noxious thermal stimulus using a plantar tester (BME-410C, Institute of Biological Medicine, Academy of Medical Science, China). Briefly, rats were placed in a clear plastic chamber (220 mm *∗* 110 mm *∗* 280 mm) and allowed to acclimatize for 30 min. A radiant heat stimulus was positioned under the glass floor directly beneath the right hind paw. When the rat withdrew its hind paw, we pressed the button to stop the heat stimulus, and the time was recorded as thermal PWL. Screening pain threshold before experiment: the rats with the PWL higher than 20 s or lower than 14 s were excluded from the experiment. A 30 s cut-off was used to prevent tissue injury. PWL was established by averaging the latency of 3 tests with a 5 min interval between each test. PWL was measured pre-CFA/normal saline injection and at D0 (after CFA injection), D1 (after MA), D7 (after MA), and D21 (after MA) at 14 o'clock to 16 o'clock.

### 2.5. Measurement of Hind Paw Swelling

The swelling of rat's right hind paw was measured by volumetric method [11] with a self-made foot volume meter. The measured time was the same as PWL. The hind paw was immersed in a chamber containing PBS up to the boundary between hairy and nonhairy skin. The volume displacement represented the hind paw swelling and was determined by two observers. Paw volume was measured twice before CFA or NS injection (as basal paw volume) and at days 1, 7, and 21 after CFA or NS injection.

### 2.6. MA Treatment

Rats were immobilized in a holder and acupuncture needles (0.35 mm in diameter and 25 mm in length) were inserted to a depth of 5–7 mm at bilateral ST36 (Zusanli) acupoints. The needles were turned at a rate of 3 spins per second bidirectionally (1 spin consisted of clockwise rotation of 180° and a counterclockwise rotation of 180°) for 2 min at Deqi, mild reinforcing and attenuating. The needles were manipulated every 5 min for a 30 min session. The manipulations were performed by the same person at 13 o'clock to 14 o'clock using metronome to keep the rhythm. MA treatment was given once a day for 7 consecutive days (day 1–day 7 after CFA injection) and then given every other day (day 8–day 21 after CFA injection), for a total of 14 sessions. In order to ensure the stability and repeatability of the manipulations, the operator practiced manipulation repeatedly at the ATP-II acupuncture manipulation parameter tester (which was manufactured by Shanghai University of Traditional Chinese Medicine Shang Xin Medical Technology Company) before and during the experiment. NS group and CFA group underwent grasping and fixation similar to CFA + MA group.

### 2.7. Sample Collection

**Figure 1:**
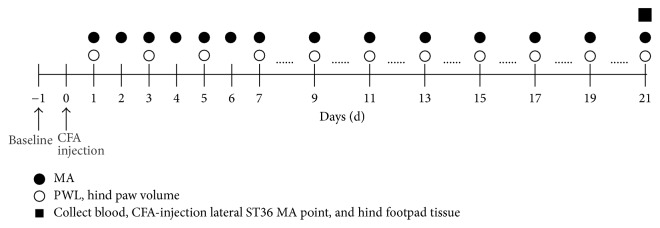
Outline of the experimental protocol.

The hair on the right legs was removed from the skin using electronic hair clipper at 1 day before sample collection. After PWL measurement at D21 after CFA injection, rats were anesthetized with chloral hydrate (35 mg/kg, i.p.). Then the blood, local tissues in right ST36, and right hind footpad tissue were collected. The blood samples by abdominal aortic method was placed for 2 h under room temperature and then were centrifuged for 2000 rpm at 4°C for 10 min to get the serum. After blood collection, the tissues located in the right ST36 (1 cm in diameter and 0.5 cm thick, consisting of skin and subcutaneous and muscle tissues) and hind footpad tissue (skin and muscle) were collected immediately with an scapel. Next all tissues were triturated into homogenate with liquid nitrogen. 4°C and 2000 r/min for 10 min centrifugation was performed to get supernatants. The serum and supernatants were stored at −80°C before detecting the NEI common signaling molecules. The outline of the experimental protocol is summarized in Figure 1.

### 2.8. Liquid Chip, RIA, and ELISA Detection

55 NEI common signaling molecules in rats serum, supernatants form the right ST36 acupoint, and hind footpad tissue were detected in this experiment, including 13 neurotransmitters or neuropeptides, 18 endocrine hormones, and 24 cytokines. Due to the limitation of the detection range of current detecting techniques, only one technique cannot detect all signaling molecules completely, so we combined liquid chip, RIA, and ELISA to detect these signaling molecules.

RIA and ELISA were conducted by Beijing Sinouk Institute of Biological Technology. Rat pituitary, rat stress hormone, rat thyroid, and rat neuropeptide liquid chip kit (Germany, Merck Millipore) were conducted by Beijing Institute of Hepatology. Bio-Plex Pro™ Rat Cytokine 24-plex Assay liquid chip kit (American, Bio-Rad) was conducted by Beijing Jian Yuan Wei Ye Technology Co., Ltd. Signaling molecules detection was carried out strictly according to the manufacturer's recommendations. The classification and detection methods of the signaling molecules were shown in Table 1.

### 2.9. Data Analysis

The data about PWL and hind paw swelling were analyzed by statistical analysis method. The data about changes of common signaling molecules in NEI network of 3 kinds of samples induced by MA on day 21 were analyzed by 2 methods, including statistical analysis and complex network analysis.

#### 2.9.1. Statistical Analysis

All statistical tests were conducted using SPSS 19.0 (SPSS Inc, Chicago, IL, USA) software. All statistical data were presented as the mean ± standard error. A *P* value < 0.05 was considered to represent statistical significance.

PWL and hind paw volume data were analyzed using repeated measures analysis of variance (ANOVA), followed by Student-Newman-Keuls test which was used for post hoc analysis for differences between groups. If Mauchly's test of sphericity was not satisfied, One-Way ANOVA followed by LSD or Dunnett's T3 post hoc test were conducted. If data were not normally distributed or violated an assumption of a statistical test, they were transformed using commonly accepted methods or analyzed with a nonparametric test.

Signaling molecules were analyzed using One-Way ANOVA the same as above. The signaling molecules with statistical significance between groups (CFA group compared with NS group or CFA + EA group) were identified as the possible key signaling molecules in MA modulating the NEI network. All figures were generated using GraphPad Prism (GraphPad Software, La Jolla, CA).

#### 2.9.2. Complex Network Analysis

For more direct and visual analysis of the NEI changes induced by MA, all of the 55 common signaling molecules in the samples were analyzed by complex network methods. The complex network analysis method consists of 3 steps. In Step 1, based on the detection results, correlation coefficients between 55 NEI network signaling molecules of the serum, supernatants form the right ST36 point, and hind footpad tissue were calculated by Pearson correlation coefficient formula, and the correlation matrix was constructed by MATLAB software (Natick, America). In Step 2, the thresholds were used to filter the signaling molecules of the correlation coefficients *∈* [−1, −0.8], [0.8, 1]. In Step 3, screened signaling molecules were sorted by 3 complex network methods (i.e., node strength correlations, node degree, and node clustering coefficient) that could measure the importance of nodes in the network. Two or more methods in The first three nodes obtained by analysis with 2 or 3 methods mentioned above were considered as the key signaling molecules in the NEI network. The 3 complex network methods were as follows. ① Node strength correlations: in complex network theory, signal molecules were viewed as nodes; the strength correlations were sum of absolute values of correlation coefficients of each node. Nodes were sorted from high to low by strength correlations, a node of higher strength correlations meaning it was more important in the network. ② Node degree: the degree was the number of edges connected to each node. Nodes were sorted from large to small by degree, a node of larger degree meaning it was more important in the network. ③ Node clustering coefficient: it represented the possibility of connections between the other nodes that were connected to this one node. Nodes were sorted from low to high by the clustering coefficient, a node of lower strength correlations meaning the other nodes are with low possibility of connections except this one node, this also suggested that the node was important in the network.

## 3. Results

### 3.1. Analgesia Effect of MA on CFA-Induced Hyperalgesia

**Figure 2:**
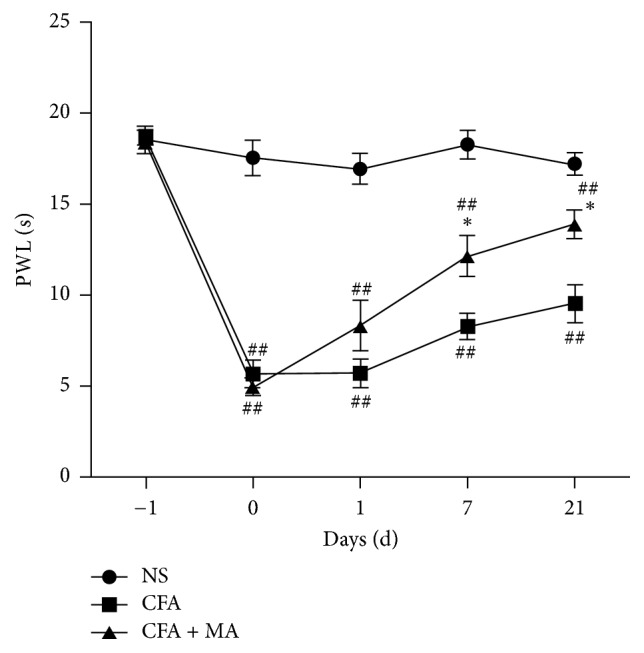
*Effect of MA on thermal hyperalgesia*. It shows that analgesic effect of MA can be detected on day 7 and day 21 after treatment. *N* = 7 per group. ^##^*P* < 0.01, CFA versus NS, or CFA + MA versus NS. ^*∗*^*P* < 0.05, CFA + MA versus CFA.

As shown in Figure 2, there were no differences in PWL under baseline condition (before CFA injection) among the three groups (NS: 18.62 ± 2.14 s, CFA: 18.75 ± 1.26 s, and CFA + MA: 18.47 ± 1.94 s) (*P* > 0.05). The PWL was significantly lower (*P* < 0.01) in CFA and CFA + MA groups (CFA: 5.70 ± 2.25 s, CFA + MA: 4.98 ± 1.47 s) on day 0 after CFA injection than in the NS group (17.62 ± 2.76 s). On day 7 and day 21, in CFA + MA group, PWL was increased significantly after MA (12.20 ± 3.37 s, 13.95 ± 2.73 s on days 7 and 21, resp.) compared with CFA group (8.32 ± 2.18 s, 9.60 ± 3.38 s on days 7 and 21, resp.) (*P* < 0.05). It indicated that MA had an analgesic effect on inflammatory pain in CFA rats.

### 3.2. The Anti-Inflammatory Effect of MA on CFA-Induced Hind Paw Swelling

**Figure 3:**
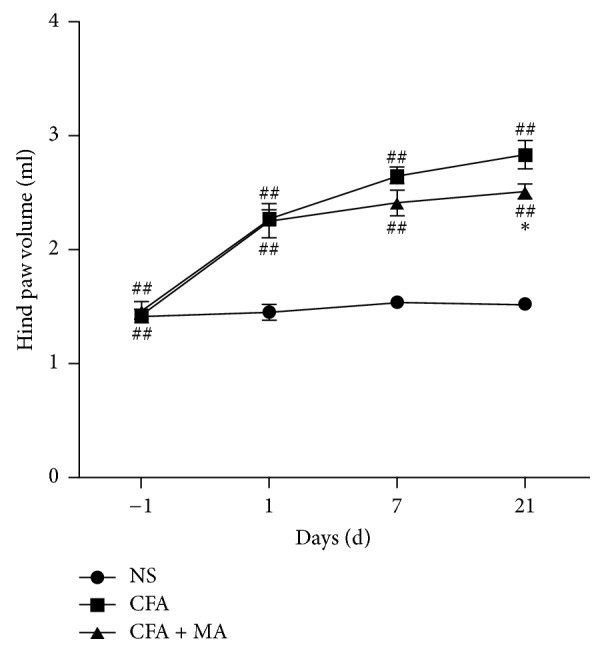
*Effect of MA on hind paw swelling*. A significant anti-inflammatory effect of MA on hind paw swelling in CFA rats was observed on day 21. *N* = 7 per group. ^##^*P* < 0.01, CFA versus NS, or CFA + MA versus NS. ^*∗*^*P* < 0.05, CFA + MA versus CFA.

As shown in Figure 3, there were no differences in hind paw swelling among the three groups prior to CFA injections (NS: 1.42 ± 0.15 ml, CFA: 1.42 ± 0.14 ml, and CFA + MA: 1.47 ± 0.19 ml) (*P* > 0.05). The right hind paw of rats swelled significantly (*P* < 0.01) in CFA and CFA + MA groups (CFA: 2.27 ± 0.19 ml, 2.65 ± 0.18 ml, 2.84 ± 0.30 ml; CFA + MA: 2.26 ± 0.36 ml, 2.42 ± 0.27 ml, 2.51 ± 0.17 ml, resp.) on days 1, 7, and 21 after CFA injections compared with the NS group (1.46 ± 0.16 ml, 1.54 ± 0.13 ml, 1.53 ± 0.13 ml, resp.). However, the hind paw swelling significantly decreased (*P* < 0.05) in CFA + MA group (2.51 ± 0.17 ml) on day 21 compared with CFA group (2.84 ± 0.30 ml), when MA treatment persisted. These results suggested that MA could alleviate the CFA-induced hind paw swelling.

### 3.3. Changes of the Common Signaling Molecules in NEI Network during MA Treatment

#### 3.3.1. The Changes of Common Signaling Molecules Based on Statistical Analysis

**Figure 4:**
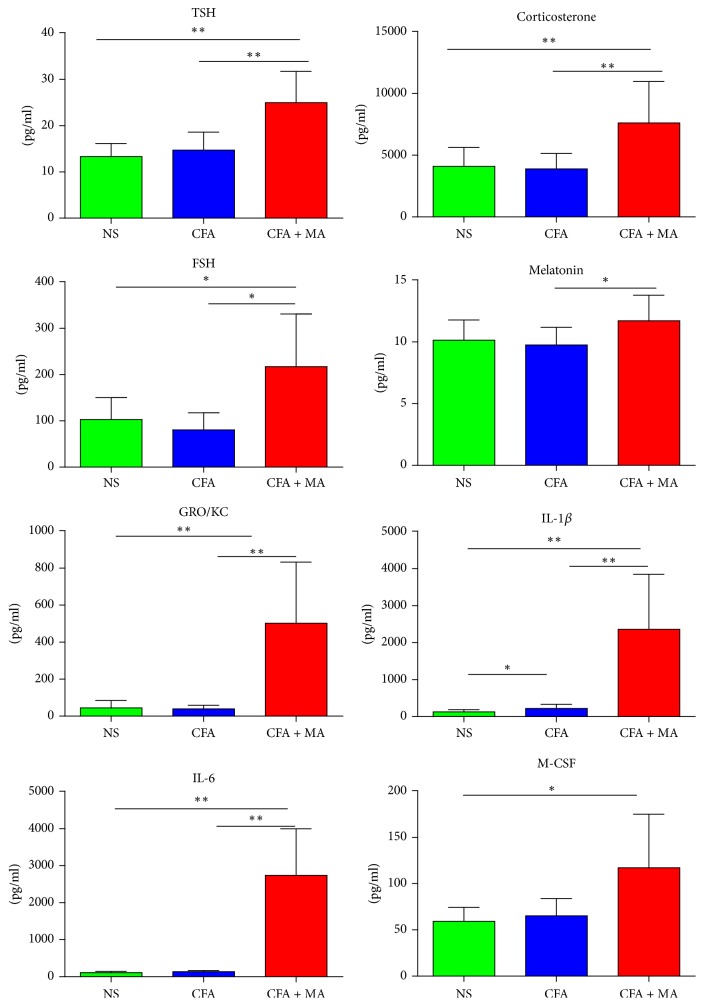
*Significant changes of the common signaling molecules in the ST36 acupoint induced by MA*. A significant statistical differences in common signaling molecules in ST36 acupoint of CFA rats induced by MA was observed on day 21. *N* = 7 per group. ^*∗*^*P* < 0.05, CFA + MA versus CFA, CFA versus NS, or CFA + MA versus NS. ^*∗∗*^*P* < 0.01, CFA + MA versus CFA, CFA + MA versus NS.

**Figure 5:**
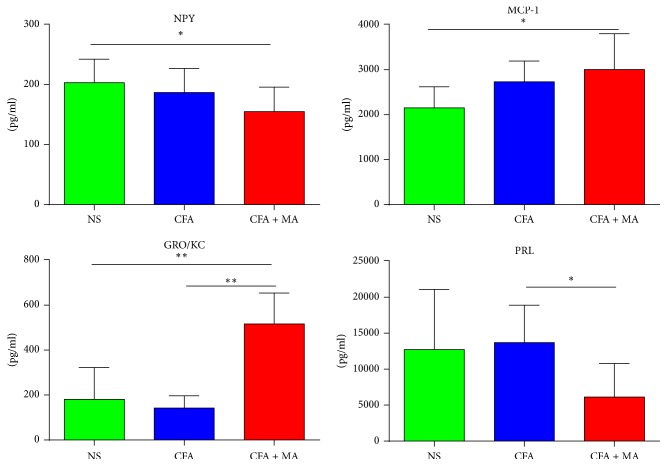
*Significant changes of common signaling molecules in serum induced by MA*. A significant statistical differences in common signaling molecules in serum of CFA rats induced by MA was observed on day 21. *N* = 7 per group. ^*∗*^*P* < 0.05, CFA + MA versus CFA, or CFA + MA versus NS. ^*∗∗*^*P* < 0.01, CFA + MA versus CFA, or CFA + MA versus NS.

**Figure 6:**
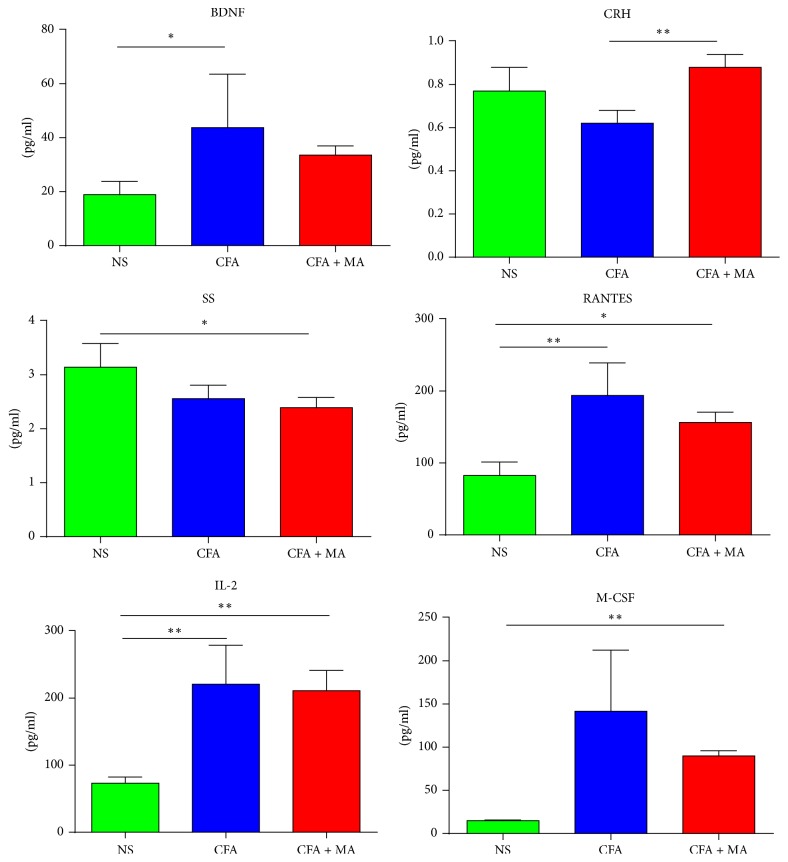
*Significant changes of common signaling molecules in the hind footpad tissue induced by MA*. Significant statistical differences in common signaling molecules in hind footpad tissue of CFA rats induced by MA was observed on day 21. *N* = 7 per group. ^*∗*^*P* < 0.05, CFA versus NS, or CFA + MA versus NS. ^*∗∗*^*P* < 0.01, CFA + MA versus CFA, CFA versus NS, or CFA + MA versus NS.

The data with significant statistical differences about the common signaling molecules in the ST36 point, serum, and hind footpad tissue among the three groups on day 21 were separately shown in Figures 4, 5, and 6. The data without statistical difference among the three groups were not shown in this part. As shown in Figure 4, in the ST36 point, cytokine IL-1*β* levels were increased in both CFA (222.79 ± 111.217 pg/ml) and CFA + MA (2361.40 ± 1484.84 pg/ml) groups. In addition, the levels of endocrine hormone TSH, corticosterone, FSH, melatonin (24.99 ± 6.78 pg/ml, 7620.97 ± 3364.50 pg/ml, 217.25 ± 113.93 pg/ml, and 11.71 ± 2.08 pg/ml, resp.), and cytokine IL-6, GRO/KC (2738.84 ± 1260.99 pg/ml, 502.40 ± 330.44 pg/ml, resp.) were upregulated after MA. As shown in Figure 5, in the serum, the level of cytokine GRO/KC was upregulated and endocrine hormone PRL was downregulated after MA (515.95 ± 137.45 pg/ml, 6125.71 ± 4661.44 pg/ml, resp.). As shown in Figure 6, in the hind footpad tissue, neurotransmitter BDNF and cytokine, namely, RANTES and IL-2 (193.84 ± 45.32 pg/ml, 220.87 ± 57.96 pg/ml, resp.), level were increased in the CFA group (43.77 ± 19.77 pg/ml). In addition, the level of endocrine hormone CRH was upregulated after MA (0.88 ± 0.06 pg/ml). These signaling molecules which were identified as statistically significant may be the possible key signaling molecules of NEI network.

#### 3.3.2. The Changes of Common Signaling Molecules Based on Complex Network Analysis

**Figure 7:**
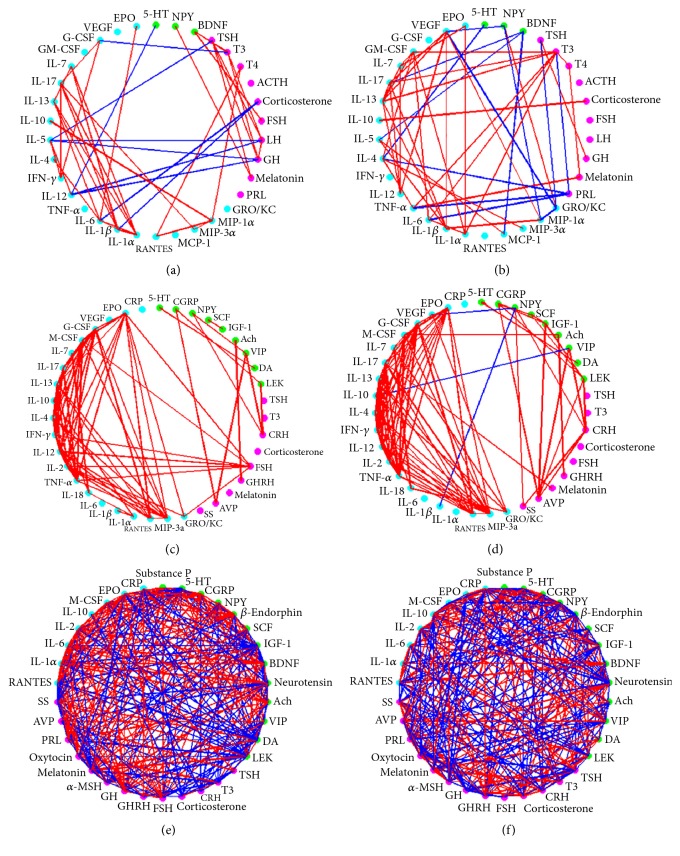
Common signaling moleculars association network in different samples in groups on day 21 (correlation coefficients *∈* [−1, −0.8], [0.8, 1]). Common signaling moleculars association network in serum in CFA group (a) or in CFA + MA group (b). Common signaling moleculars association network in ST36 acupoint in CFA group (c) or in CFA + MA group (d). Common signaling moleculars association network in hind foot pad tissue in CFA group (e) or in CFA + MA group (f). Green dots represent neuropeptide or neurotransmitter, pink dots represent hormone, and blue dots represent cytokines. Red lines represents positive correlation between the molecules; blue lines represent negative correlation between the molecules. The thicker line represents greater correlation coefficient; the thinner line represents smaller correlation coefficient.

Correlation coefficients between the common signaling molecules of the NEI network in ST36 acupoint, serum, and lateral hind footpad tissue were calculated by Pearson correlation coefficient formula, and signaling moleculars association network (see Figure 7) was constructed based on the signaling molecules chosen by correlation coefficients *∈* [−1, −0.8], [0.8, 1]. Then the selected signaling molecules were sorted by node strength correlations, node degree, and node clustering coefficient (see Tables 2–7.). The first three nodes obtained by analysis with 2 or 3 methods mentioned above were considered as the key signaling molecules in the NEI network (see Table 8).

## 4. Discussion

In this study, the results showed that the PWL obviously decreased and hand paw swelling increased after CFA injections; MA could significantly increase the PWL and decrease hand paw swelling of the CFA rats; it indicated that MA had anti-inflammatory and antinociceptive effect on inflammatory pain in CFA rats. This is consistent with other studies [12–14].

The analysis of statistical results shows that, in CFA group, some common signaling molecules of NEI network in hind foot pad tissue were increased compared with NS group, including proinflammatory cytokines RANTES, IL-2, neuropeptide BDNF, indicating that hind footpad tissue after CFA injection was in inflammatory condition. Present studies indicated many similarities regarding the immunological changes and pathologic mechanisms existed between the CFA model and human rheumatoid arthritis (RA). So the CFA model is the widely used animal model for researching mechanisms and therapies of human RA [15]. Cytokines play an important role in pathogenesis of RA, for example, IL-2 could promote inflammatory response, activate macrophages and neutrophils, and inhibit Th2 lymphocyte proliferation in RA [16]. Other studies have found that the chemokine RANTES, secreted by monocytes/macrophages in the synovia of RA patients, was significantly increased [17] and could promote osteoclast formation [18], leading to increased inflammation. These were consistent with our results. The increased CRH in hind footpad tissue induced by MA should be further investigated.

Results about the common signaling molecules of NEI network in serum in CFA rats showed that GRO/KC increased and PRL decreased after MA treatment; that still needs to be further studied. Results about the common signaling molecules of NEI network in ST36 acupoint in CFA rats showed that MA induced the increasing expression of some hormones (TSH, melatonin, corticosterone, and FSH), proinflammatory cytokines (IL-1*β*, IL-6), and chemokine GRO/KC. As MA is a kind of noxious stimulation, so these changes of signaling molecules in ST36 acupoint maybe the normal responses to noxious stimulation and specific responses to MA. Zhang et al. proposed the concept of Neural Acupuncture Unit: acupuncture could excite nerve in the local acupoint and could also activate the cells closely connected with the nerve, so as to promote the release of neurotransmitters, hormones, and cytokines, and then transfer the acupuncture information [19]. Hi-Joon Par found that some neural and immune pathways, such as MAPK, B-cell receptor, T-cell receptor, and Toll-like receptor, in the local acupoint were involved in the anti-inflammatory and antinociceptive effect of acupuncture on inflammatory pain in CFA rats [20]. These findings support our results. The changes of these molecules may play a key role in the production and transmission of acupuncture information [21]. It still needs further research in the future.

The analysis of complex network results shows that, in serum, IL-1*β*, IL-6, and IL-1*α* were the key signaling molecules in CFA rats, and T3, IL-13, and VEGF were the key signaling molecules in CFA + MA rats. Moreover, the key signaling molecules in hind footpad tissue of CFA rats were EPO, SP, and IL-1*α* and in CFA + MA rats were substance P, neurotensin, and oxytocin. These indicate that the key signaling molecules we acquired were consistent with the pathogenesis of RA and the analgesic effect and anti-inflammatory mechanism of MA. Some researches reported that IL-2, IL-12, IL-1*α*, IL-1*β*, IL-6, and TNF-*α* could induce acute phase reaction, stimulate the growth and differentiation of hematopoietic precursor cells, promote the proliferation of synovial fibroblasts, and cause joint damage [22] in the pathogenesis of RA. IL-13, produced by activated Th2 cells, had anti-inflammatory and immunomodulatory effects [23]. Therefore, these signaling molecules may play a key role in acupuncture regulating the NEI network but require further experimental confirmation. Other key signaling molecules identified, such as VEGF, EPO, SP, NT, and OT, need further experiments to explain the result. In the ST36 acupoint, the results of complex network showed that the key signaling molecules after MA were IFN-*γ*, MIP-3*α*, and TNF-*α*, and the key signaling molecules identified by statistical methods included IL-1*β*, IL-6, and GRO/KC, which belong to proinflammatory cytokines or chemokines, so we hypothesize that acupuncture, as a physical stimulus, may cause an inflammatory reaction in the local acupoint, amplify the acupuncture information in cascade, and act on the NEI network, eventually producing acupuncture effects. The inflammatory response induced by acupuncture may be the starting point of acupuncture effect. In the future we will further research this part.

In this study, we detected 55 kinds of common signaling molecules of NEI network in three parts of CFA rats, including serum, ST36 acupoint, and hind footpad tissue. Since some signaling molecules in the three parts failed to be detected by the measurement techniques, the types and numbers of the signaling molecules detected in three parts were different. So it may affect the analysis results.

## 5. Conclusions

In conclusion, this study shows that MA has obvious analgesic and anti-inflammatory effects on CFA rats with inflammatory pain; the key signaling molecules of ST36 acupoint, serum, and hind footpad tissue acquired by statistical and complex network methods were all consistent with the pathogenesis of RA and the analgesic effect and anti-inflammatory mechanism of MA; these key signaling molecules in the three parts may play an important role in MA modulating NEI network; it still needs to be further studied in the future.

## Figures and Tables

**Table 1:** Classification and detection methods of 55 NEI common signaling molecules.

Number	Signaling molecule	Classification	Detection method
(1)	Substance P	N	Liquid chip
(2)	5-Hydroxytryptamine (5-HT)	N	RIA/ELISA
(3)	Calcitonin gene related peptide (CGRP)	N	RIA/ELISA
(4)	Neuropeptide Y (NPY)	N	RIA/ELISA
(5)	Acetylcholine (Ach)	N	Liquid chip
(6)	Vasoactive intestinal peptide (VIP)	N	Liquid chip
(7)	Dopamine (DA)	N	Liquid chip
(8)	Leu-Enkephalin (LEK)	N	Liquid chip
(9)	*β*-Endorphin	N	Liquid chip
(10)	Stem cell factor (SCF)	N	RIA/ELISA
(11)	Insulin-like growth factor-1 (IGF-1)	N	RIA/ELISA
(12)	Brain derived neurotrophic factor (BDNF)	N	Liquid chip
(13)	Neurotensin	N	Liquid chip
(14)	Thyroid stimulating hormone (TSH)	E	Liquid chip
(15)	Triiodothyronine (T3)	E	Liquid chip
(16)	Thyroxine (T4)	E	Liquid chip
(17)	Corticotropin releasing hormone (CRH)	E	RIA/ELISA
(18)	Adreno-cortico-tropic-hormone (ACTH)	E	Liquid chip
(19)	Corticosterone	E	Liquid chip
(20)	Follicle-stimulating hormone (FSH)	E	Liquid chip
(21)	Luteinizing hormone (LH)	E	Liquid chip
(22)	Growth-hormone-releasing hormone (GHRH)	E	RIA/ELISA
(23)	Growth hormone (GH)	E	Liquid chip
(24)	Somatostatin (SS)	E	Liquid chip
(25)	*α*-MSH	E	Liquid chip
(26)	Melatonin	E	Liquid chip
(27)	Orexin A	E	Liquid chip
(28)	Oxytocin	E	Liquid chip
(29)	Prolactin (PRL)	E	Liquid chip
(30)	Arginine Vasopressin (AVP)	E	RIA/ ELISA
(31)	Erythropoietin (EPO)	E	Liquid chip
(32)	Growth-related oncogene/keratinocyte-derived chemokines (GRO/KC)	C	Liquid chip
(33)	Macrophage inflammatory protein-1*α* (MIP-1*α*)	C	Liquid chip
(34)	Macrophage inflammatory protein-3*α* (MIP-3*α*)	C	Liquid chip
(35)	Monocyte chemotactic protein 1 (MCP-1)	C	Liquid chip
(36)	Regulated on activation, normal T cell expressed and secreted (RANTES)	C	Liquid chip
(37)	Interleukin 1 alpha (IL-1*α*)	C	Liquid chip
(38)	Interleukin 1 beta (IL-1*β*)	C	Liquid chip
(39)	Interleukin 6 (IL-6)	C	Liquid chip
(40)	Interleukin 18 (IL-18)	C	Liquid chip
(41)	Tumor necrosis factor alpha (TNF-*α*)	C	Liquid chip
(42)	Interleukin 2 (IL-2)	C	Liquid chip
(43)	Interleukin 12 (IL-12)	C	Liquid chip
(44)	Interferon gamma (IFN-*γ*)	C	Liquid chip
(45)	Interleukin 4 (IL-4)	C	Liquid chip
(46)	Interleukin 5 (IL-5)	C	Liquid chip
(47)	Interleukin 10 (IL-10)	C	Liquid chip
(48)	Interleukin 17 (IL-17)	C	Liquid chip
(49)	Interleukin 13 (IL-13)	C	Liquid chip
(50)	Interleukin 7 (IL-7)	C	Liquid chip
(51)	Granulocyte-macrophage colony stimulating factor (GM-CSF)	C	Liquid chip
(52)	Macrophage colony-stimulating factor (M-CSF)	C	Liquid chip
(53)	Granulocyte-colony stimulating factor (G-CSF)	C	Liquid chip
(54)	Vascular endothelial growth factor (VEGF)	C	Liquid chip
(55)	C-reactive protein (CRP)	C	Liquid chip

N: neurotransmitter or neuropeptide; E: endocrine hormone; C: cytokine.

**Table 2:** Common signaling moleculars in serum of CFA group sorted by complex network analysis.

Node strength correlations	Molecular sorting	Node degree	Molecular sorting	Node clustering coefficient	Molecular sorting
5.364006319	IL-1*β*	6	IL-1*β*	0	5-HT
5.120790119	IL-6	6	IL-6	0	NPY
4.464794999	IL-1*α*	5	LH	0	BDNF
4.356832829	LH	5	GH	0	ACTH
4.356832829	GH	5	IL-1*α*	0	FSH
4.177256544	IL-17	5	IL-5	0	Melatonin
4.09114429	IL-5	5	IL-17	0	PRL
3.6458827	IL-13	4	TSH	0	GRO/KC
3.53653472	TSH	4	T3	0	MIP-3*α*
3.400877137	IL-12	4	MIP-1*α*	0	MCP-1
3.364263888	MIP-1*α*	4	IL-12	0	TNF-*α*
3.238781977	T3	4	IL-13	0	IL-4
2.605926586	G-CSF	3	IFN-*γ*	0	IL-10
2.5478666	IFN-*γ*	3	IL-7	0	GM-CSF
2.524251392	IL-7	3	G-CSF	0	VEGF
1.809686502	Melatonin	2	BDNF	0	EPO
1.728692888	Corticosterone	2	T4	0.166666667	T3
1.711474914	MIP-3*α*	2	Corticosterone	0.166666667	MIP-1*α*
1.675529628	RANTES	2	Melatonin	0.2	IL-17
1.638782764	T4	2	MIP-3*α*	0.266666667	IL-6
1.60762883	BDNF	2	RANTES	0.333333333	IFN-*γ*
0.918065642	5-HT	1	5-HT	0.333333333	G-CSF
0.865532438	NPY	1	NPY	0.466666667	IL-1*β*
0.837856344	IL-10	1	FSH	0.5	TSH
0.834973817	IL-4	1	IL-4	0.5	IL-12
0.816730828	EPO	1	IL-10	0.5	IL-5
0.806908726	FSH	1	EPO	0.6	LH
0	ACTH	0	ACTH	0.6	GH
0	PRL	0	PRL	0.6	IL-1*α*
0	GRO/KC	0	GRO/KC	0.833333333	IL-13
0	MCP-1	0	MCP-1	1	T4
0	TNF-*α*	0	TNF-*α*	1	Corticosterone
0	GM-CSF	0	GM-CSF	1	RANTES
0	VEGF	0	VEGF	1	IL-7

**Table 3:** Common signaling moleculars in serum of CFA + MA group sorted by complex network analysis.

Node strength correlations	Molecular sorting	Node degree	Molecular sorting	Node clustering coefficient	Molecular sorting
6.793769656	T3	8	T3	0	5-HT
6.1279229	IL-13	7	IL-13	0	TSH
6.125755358	VEGF	7	VEGF	0	T4
5.345425536	IL-17	6	IL-17	0	ACTH
5.058280921	GM-CSF	6	GM-CSF	0	Corticosterone
4.32642674	IL-1*β*	5	IL-1*β*	0	FSH
4.23929481	IL-4	5	IL-4	0	LH
3.653747121	MIP-1*α*	4	PRL	0	GH
3.536793987	GRO/KC	4	GRO/KC	0	Melatonin
3.520423163	IL-12	4	MIP-1*α*	0	MIP-3*α*
3.472198204	IL-7	4	IL-1*α*	0	RANTES
3.442092944	PRL	4	IL-6	0	IFN-*γ*
3.437908006	IL-1*α*	4	TNF-*α*	0	IL-10
3.343295878	IL-6	4	IL-12	0	G-CSF
3.326627841	TNF-*α*	4	IL-7	0	EPO
2.790223761	NPY	3	NPY	0.1	IL-4
2.628870992	IL-5	3	BDNF	0.25	T3
2.541007235	BDNF	3	IL-5	0.333333333	BDNF
1.831565026	T4	2	TSH	0.333333333	TNF-*α*
1.810788756	MCP-1	2	T4	0.333333333	IL-5
1.743848565	Melatonin	2	Melatonin	0.333333333	IL-7
1.73742207	EPO	2	MCP-1	0.333333333	GM-CSF
1.668735302	TSH	2	EPO	0.4	IL-1*β*
0.860173882	Corticosterone	1	5-HT	0.4	IL-17
0.860173882	IL-10	1	Corticosterone	0.428571429	IL-13
0.831360077	GH	1	GH	0.428571429	VEGF
0.825950091	MIP-3*α*	1	MIP-3*α*	0.5	PRL
0.811762933	5-HT	1	IL-10	0.5	GRO/KC
0	ACTH	0	ACTH	0.5	IL-1*α*
0	FSH	0	FSH	0.666666667	MIP-1*α*
0	LH	0	LH	0.666666667	IL-6
0	RANTES	0	RANTES	0.666666667	IL-12
0	IFN-*γ*	0	IFN-*γ*	1	NPY
0	G-CSF	0	G-CSF	1	MCP-1

**Table 4:** Common signaling moleculars in ST36 acupoint of CFA group sorted by complex network analysis.

Node strength correlations	Molecular sorting	Node degree	Molecular sorting	Node clustering coefficient	Molecular sorting
10.72648569	IL-2	12	IL-2	0	5-HT
9.92879842	IL-12	11	IL-12	0	CGRP
9.756829776	IL-13	11	IL-13	0	SCF
9.692100786	M-CSF	11	M-CSF	0	IGF-1
8.865265516	EPO	10	TNF-*α*	0	Ach
8.864817973	IL-4	10	IL-4	0	DA
8.756035321	TNF-*α*	10	EPO	0	LEK
8.190175267	MIP-3*α*	9	MIP-3*α*	0	TSH
7.868172048	G-CSF	9	G-CSF	0	T3
6.823300188	IFN-*γ*	8	IFN-*γ*	0	Corticosterone
6.281832099	RANTES	7	FSH	0	GHRH
6.133378999	FSH	7	RANTES	0	Melatonin
5.071780915	IL-17	6	IL-17	0	AVP
2.725513459	IL-18	3	VIP	0	SS
2.697281121	CRH	3	CRH	0	IL-1*β*
2.695360587	VIP	3	GRO/KC	0	IL-6
2.670612454	GRO/KC	3	IL-1*α*	0	VEGF
2.614138569	IL-10	3	IL-18	0	CRP
2.549577048	IL-1*α*	3	IL-10	0.333333333	VIP
1.798304838	LEK	2	CGRP	0.333333333	CRH
1.758387897	CGRP	2	NPY	0.333333333	IL-1*α*
1.757512896	NPY	2	LEK	0.666666667	IL-18
1.711380393	AVP	2	AVP	0.666666667	IL-2
1.704535511	IL-7	2	IL-7	0.666666667	IL-10
0.999281664	5-HT	1	5-HT	0.688888889	EPO
0.999281664	DA	1	SCF	0.745454545	M-CSF
0.870807119	IL-1*β*	1	IGF-1	0.755555556	TNF-*α*
0.863267828	Ach	1	Ach	0.761904762	FSH
0.863267828	GHRH	1	DA	0.763636364	IL-12
0.832906845	SCF	1	GHRH	0.763636364	IL-13
0.832906845	IGF-1	1	IL-1*β*	0.777777778	IL-4
0	TSH	0	TSH	0.805555556	G-CSF
0	T3	0	T3	0.821428571	IFN-*γ*
0	Corticosterone	0	Corticosterone	0.866666667	IL-17
0	Melatonin	0	Melatonin	0.888888889	MIP-3*α*
0	SS	0	SS	0.952380952	RANTES
0	IL-6	0	IL-6	1	NPY
0	VEGF	0	VEGF	1	GRO/KC
0	CRP	0	CRP	1	IL-7

**Table 5:** Common signaling moleculars in ST36 acupoint of CFA + MA group sorted by complex network analysis.

Node strength correlations	Molecular sorting	Node degree	Molecular sorting	Node clustering coefficient	Molecular sorting
10.30508457	IFN-*γ*	11	MIP-3*α*	0	5-HT
10.20967738	MIP-3*α*	11	TNF-*α*	0	Ach
10.14565087	TNF-*α*	11	IL-2	0	DA
10.14143505	EPO	11	IFN-*γ*	0	TSH
9.984491306	IL-2	11	IL-17	0	T3
9.835412633	IL-17	11	EPO	0	Corticosterone
9.586116688	IL-4	10	RANTES	0	FSH
9.58555599	IL-13	10	IL-12	0	GHRH
9.496416209	IL-12	10	IL-4	0	Melatonin
9.324953981	G-CSF	10	IL-13	0	IL-1*α*
9.322535406	RANTES	10	G-CSF	0	IL-1*β*
5.816288172	M-CSF	7	M-CSF	0	IL-6
4.447630823	IL-7	5	LEK	0	CRP
4.428353692	LEK	5	CRH	0.166666667	NPY
4.330583532	AVP	5	AVP	0.3	CRH
4.254168273	CRH	5	IL-7	0.333333333	VIP
3.726312879	CGRP	4	CGRP	0.333333333	VEGF
3.665515648	GRO/KC	4	NPY	0.4	IL-7
3.631586867	IGF-1	4	IGF-1	0.476190476	M-CSF
3.626838652	IL-10	4	GRO/KC	0.5	AVP
3.3417145	NPY	4	IL-10	0.5	IL-10
2.863080058	IL-18	3	SCF	0.6	LEK
2.733155888	SCF	3	VIP	0.666666667	GRO/KC
2.612860185	VIP	3	IL-18	0.818181818	IL-17
2.432869853	VEGF	3	VEGF	0.833333333	CGRP
1.777426407	Ach	2	Ach	0.833333333	IGF-1
1.745137778	SS	2	SS	0.890909091	MIP-3*α*
0.995761145	5-HT	1	5-HT	0.890909091	TNF-*α*
0.995761145	DA	1	DA	0.890909091	IL-2
0.909867657	GHRH	1	GHRH	0.890909091	IFN-*γ*
0.832121031	IL-1*β*	1	Melatonin	0.890909091	EPO
0.831984815	Melatonin	1	IL-1*β*	1	SCF
0	TSH	0	TSH	1	SS
0	T3	0	T3	1	RANTES
0	Corticosterone	0	Corticosterone	1	IL-18
0	FSH	0	FSH	1	IL-12
0	IL-1*α*	0	IL-1*α*	1	IL-4
0	IL-6	0	IL-6	1	IL-13
0	CRP	0	CRP	1	G-CSF

**Table 6:** Common signaling moleculars in hind footpad tissue of CFA group sorted by complex network analysis.

Node strength correlations	Molecular sorting	Node degree	Molecular sorting	Node clustering coefficient	Molecular sorting
19.10661085	EPO	20	Substance P	0.5	TSH
19.10443745	Substance P	20	5-HT	0.5	IL-10
19.09503987	IL-1*α*	20	IGF-1	0.588235294	Neurotensin
19.08824776	IL-6	20	LEK	0.607142857	CRH
19.07123329	IGF-1	20	Melatonin	0.617647059	Oxytocin
19.06447235	5-HT	20	IL-1*α*	0.631578947	Melatonin
18.50160671	LEK	20	IL-6	0.666666667	Corticosterone
18.29600965	PRL	20	EPO	0.692307692	*α*-MSH
18.29571712	IL-2	19	*β*-Endorphin	0.705263158	LEK
18.27023516	FSH	19	DA	0.772727273	NPY
18.25430456	DA	19	FSH	0.772727273	T3
18.25266497	Melatonin	19	PRL	0.782051282	CGRP
18.0674304	M-CSF	19	SS	0.8	VIP
18.04781731	*β*-Endorphin	19	RANTES	0.8	AVP
17.94864817	SS	19	IL-2	0.807017544	SS
17.94592524	RANTES	19	M-CSF	0.807017544	RANTES
16.95153766	GH	18	GH	0.831578947	5-HT
15.47470248	Oxytocin	17	Neurotensin	0.831578947	IGF-1
15.40534815	Neurotensin	17	Oxytocin	0.831578947	IL-1*α*
14.93565299	Ach	16	BDNF	0.831578947	IL-6
14.9351783	BDNF	16	Ach	0.836363636	SCF
12.16893349	*α*-MSH	13	CGRP	0.836363636	CRP
11.68002365	CGRP	13	*α*-MSH	0.842105263	Substance P
11.2866825	NPY	12	NPY	0.842105263	*β*-Endorphin
11.27743293	T3	12	T3	0.842105263	M-CSF
10.44668172	CRP	11	SCF	0.842105263	EPO
10.43829458	SCF	11	CRP	0.85620915	GH
9.545156525	GHRH	10	VIP	0.883040936	DA
9.391637867	AVP	10	GHRH	0.883040936	FSH
9.373485793	VIP	10	AVP	0.883040936	PRL
8.148967846	Corticosterone	9	Corticosterone	0.883040936	IL-2
7.229380481	CRH	8	CRH	0.883333333	BDNF
4.484931739	TSH	5	TSH	0.883333333	Ach
3.693908813	IL-10	4	IL-10	0.888888889	GHRH

**Table 7:** Common signaling moleculars in hind foot pad tissue of CFA + MA group sorted by complex network analysis.

Node strength correlations	Molecular sorting	Node degree	Molecular sorting	Node clustering coefficient	Molecular sorting
18.40435568	Substance P	20	Substance P	0.5	DA
18.40150748	Neurotensin	20	Neurotensin	0.642857143	FSH
17.63806473	Oxytocin	19	Oxytocin	0.666666667	LEK
17.60762122	PRL	19	PRL	0.69005848	Oxytocin
16.88856039	GHRH	18	NPY	0.692810458	*β*-Endorphin
16.83720135	NPY	18	*β*-Endorphin	0.7	Substance P
16.79938918	CRP	18	GHRH	0.7	Neurotensin
16.69891691	*β*-Endorphin	18	CRP	0.705128205	IL-6
16.00352427	VIP	17	5-HT	0.705882353	5-HT
15.77571079	IL-2	17	CGRP	0.705882353	Corticosterone
15.69108923	CGRP	17	VIP	0.713235294	CGRP
15.65507789	Corticosterone	17	Corticosterone	0.713450292	PRL
15.6462691	5-HT	17	IL-2	0.714285714	RANTES
15.20421904	IGF-1	16	IGF-1	0.720588235	IL-2
15.157233	*α*-MSH	16	*α*-MSH	0.722222222	Ach
15.15132027	AVP	16	AVP	0.725490196	GHRH
15.04667388	IL-10	16	IL-10	0.727272727	GH
13.98760052	SS	15	LEK	0.736263736	CRH
13.80904047	LEK	15	SS	0.738562092	NPY
13.77891314	M-CSF	15	M-CSF	0.75	BDNF
13.1712101	TSH	14	TSH	0.752380952	SS
13.1430236	CRH	14	CRH	0.758169935	CRP
13.09659812	RANTES	14	Melatonin	0.758241758	TSH
12.88753949	Melatonin	14	RANTES	0.785714286	IL-1*α*
11.9691417	IL-6	13	T3	0.79047619	M-CSF
11.905127	T3	13	IL-6	0.794117647	VIP
11.89354354	EPO	13	EPO	0.813186813	Melatonin
11.08226901	GH	12	GH	0.816666667	*α*-MSH
8.51846022	SCF	9	SCF	0.816666667	AVP
8.373711747	BDNF	9	BDNF	0.816666667	IL-10
8.142776395	Ach	9	Ach	0.833333333	SCF
7.508261048	IL-1*α*	8	DA	0.833333333	IGF-1
7.278849151	FSH	8	FSH	0.833333333	T3
7.056443065	DA	8	IL-1*α*	0.833333333	EPO

**Table 8:** Key common signaling moleculars in different samples based on complex network analysis.

	Serum	ST36 acupoint	Hind footpad tissue
CFA	IL-1*β*	IL-2	EPO
	IL-6	IL-12	Substance P
	IL-1*α*	IL-13	IL-1*α*

CFA + MA	T3	IFN-*γ*	Substance P
	IL-13	MIP-3*α*	Neurotensin
	VEGF	TNF-*α*	Oxytocin
